# Supplementary material for: Perfusion Air Culture of Precision-Cut Tumor Slices: An Ex Vivo System to Evaluate Individual Drug Response under Controlled Culture Conditions
Source: Cells. 2023 Mar 4;12(5):807. doi: 10.3390/cells12050807 (PMC10001200; doi:10.3390/cells12050807)
Supplement: Supplementary file 1 [file cells-12-00807-s001.zip › cells-2116944-supplementary.pdf]

Supplementary material

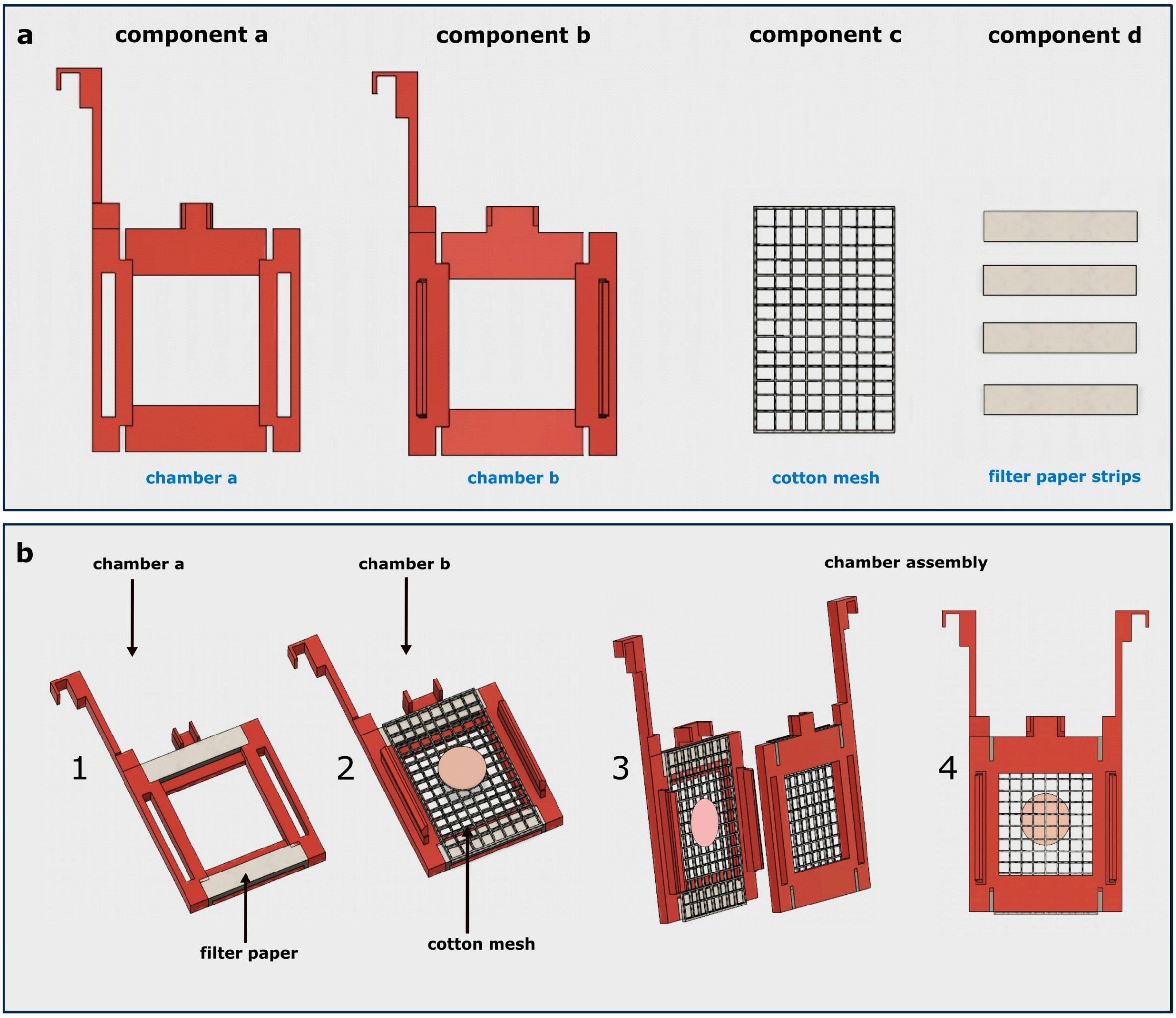

Figure S1. Structure and assembly of the perfusion air culture (PAC) system. **(a)** The 4 components a-d of the chamber. **(b)** The assembly process of the chamber with a tissue slice. The chamber consists of two main components (a and b) which can be assembled easily under sterile conditions using a click system. Both components together form the holder for the organotypic support (component c) for the tissue slices. The filter papers (component d) are fixed on the chambers (components a and b) and serve as a reservoir for the nutrient fluid. The tumor slices are inserted in between two organotypic supports and then fixed in the chamber.

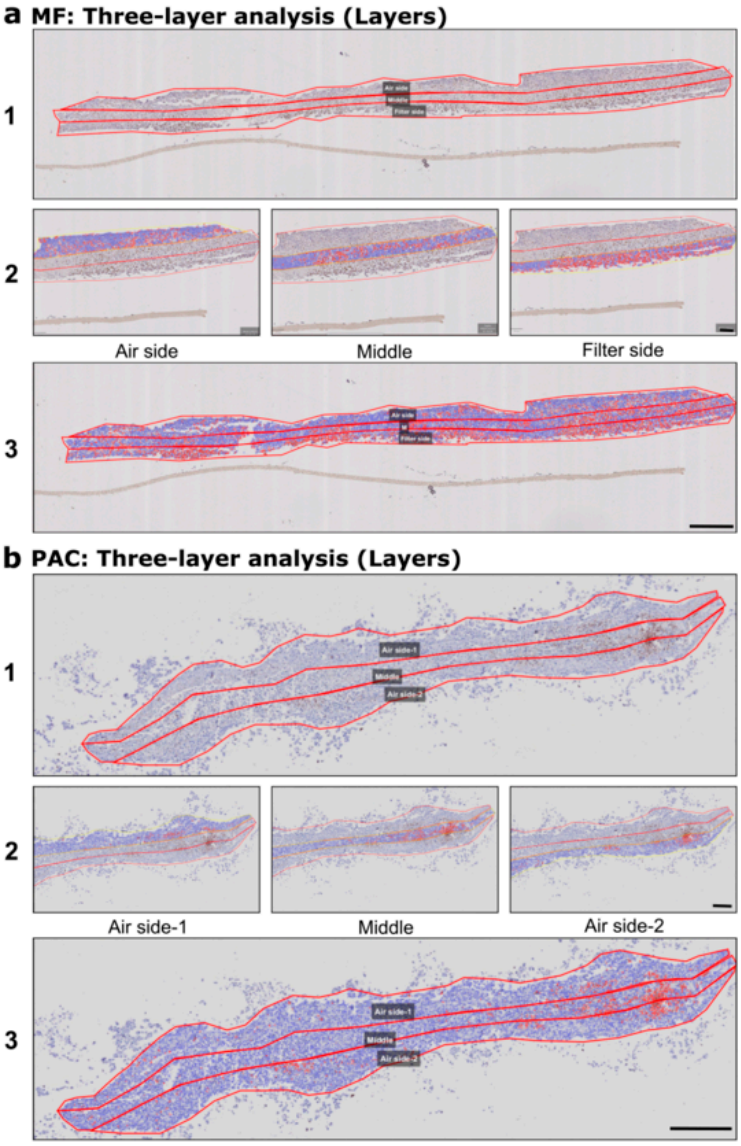

c Calculation of gradient of biomarker expression:

$$Percentage\ Difference = \frac{|Difference|}{Average} \times 100\%$$

$$For\ the\ MF\ culture\ system: \frac{|Air\ side - Filter\ side|}{avg(Air\ side + Middle + Filter\ side)} \times 100\%$$

$$For\ the\ PAC\ culture\ system: \frac{|Air\ side_1 - Air\ side_2|}{avg(Air\ side_1 + Middle + Air\ side_2)} \times 100\%$$

Figure S2. Workflow of three-layer analysis for quantification of loco-regional changes of biomarker expression in tumor slices cultivated using the Millipore filter (MF) system or the perfusion air culture (PAC) system. (a-1) Immunohistochemical staining images of whole tumor slices cultured in the MF system were split into three lengthwise layers (Filter side, Middle, Air side). (a-2) The layers were successively analyzed for biomarker expression to (a-3)quantify biomarker-positive cells in the tumor slice. Scale bar represents 400 µm. (b-1) Immunohistochemical staining images of whole tumor slices cultured in the MF system were split into three longitudinal layers (Air side-1, Middle, Air side-2). (b-2) The layers were successively analyzed for biomarker expression to (b-3) quantify biomarker-positive cells in the tumor slice. Scale bar represents 400 µm. (c) The percentage difference of biomarker-positive cells between the outer layers of the tumor slices was calculated to determine the gradient across different areas of the tumor slice.

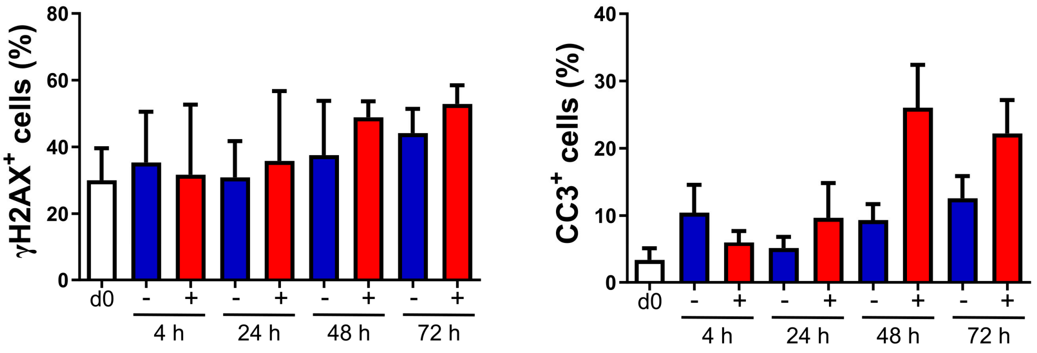

Figure S3. Quantification of the percentage of γH2AX and CC3 positive cells at different time points after cisplatin treatment of primary human ovarian tumor slices in the Millipore filter (MF) system. Primary ovarian tumor slices were cultured without cisplatin (-) or with cisplatin(+) for different time points (4 h, 24 h, 48 h and 72 h) in the Millipore filter (MF) system. Data are from experiments of four mice.

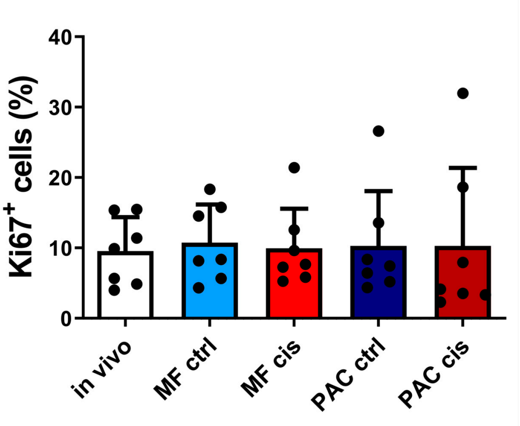

Figure S4. Quantification of the percentage of Ki67 positive cells of tumor slices cultured in the Millipore filter (MF) and perfusion air culture (PAC) systems. Primary ovarian tumor slices were cultured without cisplatin (ctrl) or with cisplatin (cis) for 3 days in the Millipore filter (MF) or the perfusion air culture (PAC) systems. In the figure, each black dot represents one patient tumor. Data are from 7 patient experiments.

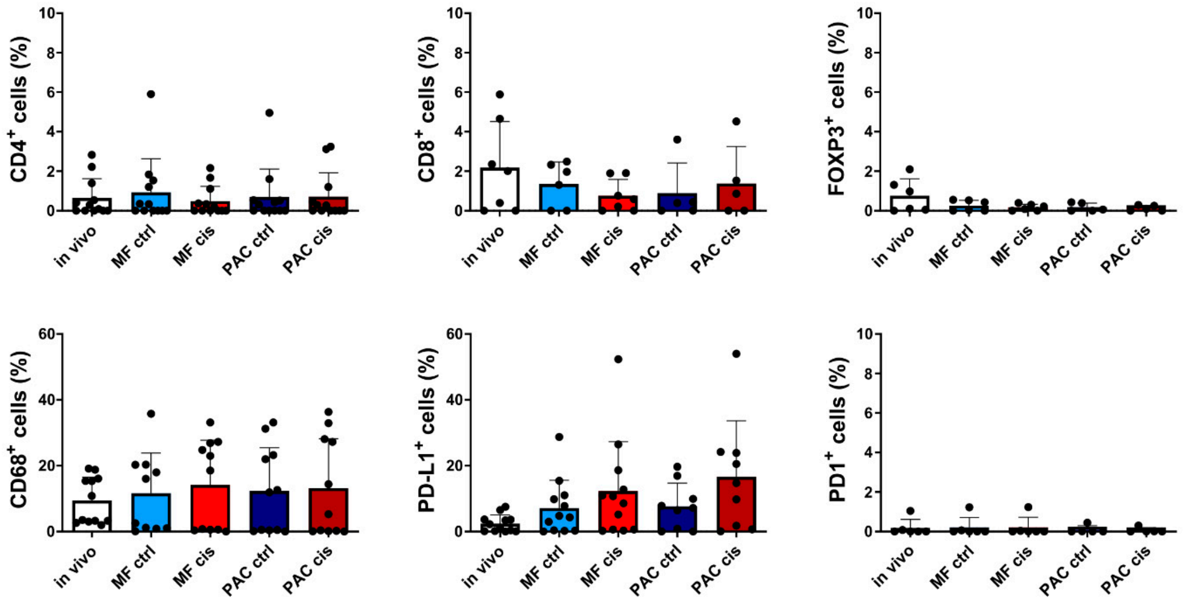

Figure S5. Quantification of the percentage of CD4, CD8, FOXP3, CD68, PD-L1 and PD1 positive cells of tumor slices in the Millipore filter (MF) and perfusion air culture (PAC) system. Primary ovarian tumor slices were cultured without cisplatin (ctrl) or with cisplatin (cis) for 3 days in the Millipore filter (MF) or the perfusion air culture (PAC) systems. Data from experiments of 6 to 12 patients. In the figures, each black dot represents one patient tumor.

Table S1. The percentage difference in biomarker expression of estrogen receptor (ER), hypoxia-inducible factor 1α (HIF1α), phosphor-Histone H2A.X (γH2Ax) and Ki67 between the filter side and air side (Millipore filter (MF)) or air side-1 and air side-2 (Perfusion air culture (PAC)) observed in slices from MCF-7 CDX after cultivation in the MF or PAC system. For the three-layer analysis of loco-regional changes, the tumor slices were lengthwise divided into three sections (MF: air side, middle, filter side; PAC: air side-1, middle, air side-2). The absolute difference in positively stained cells between the two outer tumor slice layers was divided by the sum of all three layers to calculate the percentage difference in biomarker expression. Red indicates the presence of a gradient (percentage difference of > 20%). Data shown are from the experiments of 6 different mice for d3 samples and 4 mice for d7 samples.

MCF-7 breast CDX

Marker	MCF7 Xenograft	Percentage difference in biomarker expression			
		d3		d7	
		MF	PAC	MF	PAC
ER	#1	64.95	16.14	92.77	19.51
	#2	34.63	9.52	49.53	2.86
	#3	53.42	4.38	95.68	16.83
	#4	42.79	1.14		
	#5	44.90	4.39		
	#6	68.67	5.18		
	#7			81.77	0.73
HIF1α	#1	49.55	13.44	35.14	0.18
	#2	51.90	9.64	28.04	3.11
	#3	94.71	5.75	7.37	19.97
	#4	101.51	4.25		
	#5	79.81	1.85		
	#6	42.50	5.45		
	#7			20.73	4.06
γH2Ax	#1	13.35	9.67	10.12	13.35
	#2	26.97	8.23	12.70	2.74
	#3	0.64	19.88	21.67	10.83
	#4	5.89	0.39		
	#5	6.14	2.35		
	#6	21.68	1.19		
	#7			23.15	6.97
Ki67	#1	0.39	4.74	15.72	5.43
	#2	5.51	5.63	56.37	1.10
	#3	31.79	4.82	84.59	5.42
	#4	41.15	2.94		
	#5	2.87	3.07		
	#6	20.04	0.61		
	#7			61.86	4.76

Table S2. The percentage difference in biomarker expression of hypoxia-inducible factor 1α (HIF1α), phosphor-Histone H2A.X (γH2Ax), Ki67 and cleaved-Caspase 3 (CC3) between the filter side and air side (Millipore filter (MF)) or air side-1 and air side-2 (Perfusion air culture (PAC)) observed in slices from H1437 CDX after cultivation in the MF or PAC system. For the three-layer analysis of loco-regional changes, the tumor slices were lengthwise divided into three sections (MF: air side, middle, filter side; PAC: air side-1, middle, air side-2). The absolute difference in positively stained cells between the two outer tumor slice layers was divided by the sum of all three layers to calculate the percentage difference in biomarker expression. Red indicates the presence of a gradient (percentage difference of > 20%). Data shown are from the CDX of 3 different mice.

H1437 lung CDX

Marker	H1437 Xenograft	Percentage difference in biomarker expression	
		d3	
		MF	PAC
HIF1α	#1	68.10	6.84
	#2	68.27	0.50
	#3	111.24	1.84
γH2Ax	#1	48.67	3.85
	#2	106.14	5.13
	#3	124.46	2.31
Ki67	#1	107.24	2.72
	#2	81.31	5.10
	#3	18.42	4.61
CC3	#1	92.12	2.93
	#2	59.16	0.28
	#3	92.95	4.23

Table S3. The percentage difference in biomarker expression of hypoxia-inducible factor 1α (HIF1α), phosphor-Histone H2A.X (γH2Ax), Ki67 and cleaved-Caspase 3 (CC3) between the filter side and air side (Millipore filter (MF)) or air side-1 and air side-2 (Perfusion air culture (PAC)) observed in slices from primary human ovarian cancer patients after cultivation in the MF or PAC system. For the three-layer analysis of loco-regional changes, the tumor slices were lengthwise divided into three sections (MF: air side, middle, filter side; PAC: air side-1, middle, air side-2). The absolute difference in positively stained cells between the two outer tumor slice layers was divided by the sum of all three layers to calculate the percentage difference in biomarker expression. Red indicates the presence of a gradient (percentage difference of > 20%). Data shown are from experiments of 15 patients.

Primary human ovarian cancer

Marker	Patient	Percentage difference in biomarker expression	
		d3	
		MF	PAC
HIF1α	#1	5.19	-4.39
	#2	1.36	-1.10
	#3	134.14	19.82
	#4	35.71	-10.09
	#5	-6.91	-122.34
	#6	206.05	31.02
	#7	33.63	-2.26
	#8	11.92	-17.32
	#9	-2.29	-2.32
	#10	110.14	-17.02
	#11	47.07	8.89
	#12	87.43	-12.62
	#13	125.19	7.65
	#14	24.30	13.69
	#15	-18.52	-20.41
Ki67	#1	-2.17	24.59
	#2	11.26	13.28
	#3	98.14	26.90
	#4	24.86	0.95
	#5	-15.83	-24.57
	#6	106.28	-6.22
	#7	45.67	10.50
	#8	1.40	7.24
	#9	-6.32	-1.59
	#10	113.24	9.64
	#11	103.65	5.02
	#12	59.15	-6.57
	#13	35.76	16.01
	#14	137.83	-8.63
	#15	-14.99	75.41

Table S4. The fold-changes in transcriptional biomarkers observed in slices from MCF-7 CDX, H1437 CDX and primary human ovarian tumors after cultivation in the Millipore filter (MF) or perfusion air culture (PAC) system. Red indicates significantly upregulated genes; green indicates significantly downregulated genes (p-value < 0.05).

MCF-7 breast CDX

	Process (Functional gene grouping)	Upstream Pathway/Transcription Factor	expressionCond1	ID	logFC	P.Value	adj.P.Val
PAC vs d0	apoptosis	p53/NFkB	down	TRAF1	-2.005655965	9.69293E-07	3.90948E-05
	apoptosis	p53/NFkB	down	BCL2	-1.617170634	3.56854E-06	0.000107948
	apoptosis	p53/NFkB	down	TP53AIP1	-1.778741559	6.33584E-06	0.000127773
	apoptosis	p53/NFkB	up	IER3	1.348605002	1.26163E-05	0.000218081
	apoptosis	NFkB	down	ALOX12	-1.341068964	0.000504107	0.003258684
	apoptosis	NFkB	down	NOS2A	-2.163061512	0.000585419	0.00346564
	apoptosis	p53	down	BCL2L14	-1.073715416	0.0007265	0.00399575
	cell cycle		down	TP73	-2.32592196	8.18118E-09	9.89923E-07
	cell cycle	p53/NFkB	down	TERT	-1.818063518	6.28131E-06	0.000127773
	cell cycle	p53	down	CCNG1	-1.389179567	2.40452E-05	0.000363684
	DNA damage/repair	HIF1a	down	XPA	-1.193623749	4.6059E-05	0.000619238
	DNA damage/repair	prosurvival signals and oncogenes	down	BLM	-1.298156571	5.45866E-05	0.000660498
	DNA damage/repair	p53	down	SESN1	-1.057576028	9.77233E-05	0.001074956
	p38/JNK	p38	up	PHLDA1	1.012846155	0.000248105	0.002309285
	ROS	NFkB/transcr. regulated under hypoxia	up	HIF1A	1.180760224	0.000601475	0.00346564
	UPR/ER stress	UPR target gene	down	EPHB1	-1.981288875	2.68508E-07	1.62447E-05
	UPR/ER stress	UPR target gene	down	PLA2G6	-1.019594051	0.000325182	0.002481483
MF vs d0	apoptosis	p53/NFkB	down	BCL2	-1.960611405	2.46702E-07	1.49255E-05
	apoptosis	p53/NFkB	up	IER3	1.594328692	1.41718E-06	4.28698E-05
	apoptosis	NFkB	down	BCL2L1	-1.314326066	0.000144866	0.001433282
	apoptosis	p53/NFkB	down	TP53AIP1	-1.210185287	0.000478411	0.002893389
	apoptosis	NFkB	down	NOS2A	-1.542577832	0.008384952	0.02601485
	cell cycle		down	TP73	-1.860393869	2.42734E-07	1.49255E-05
	cell cycle	p53/NFkB	down	TP63	-1.35710972	0.000150902	0.001433282
	cell cycle	p53/NFkB	down	TERT	-1.374074016	0.000165834	0.001433282
	cell cycle	p53	down	CCNA2	-1.021728382	0.005285863	0.019381499
	DNA damage/repair	p53/NFkB	down	TP53I3	-1.529581821	8.14596E-06	0.000123208
	DNA damage/repair	HIF1a	down	XPA	-1.232022901	3.18621E-05	0.000428368
	DNA damage/repair	transcr. inducable by irradiation	down	XRCC3	-1.031817735	0.000162619	0.001433282
	DNA damage/repair	prosurvival signals and oncogenes	down	BLM	-1.088122797	0.000354517	0.00225771
	p38/JNK	p38	up	PHLDA1	1.485114584	2.72921E-06	6.60468E-05
	p38/JNK	p38	up	FOSL1	1.511246682	4.05091E-06	8.16934E-05
	p38/JNK	p38	up	MAFF	1.478042154	6.70678E-06	0.000115931
	p38/JNK	p38	up	OAS1	1.19449399	0.000526071	0.002893389
	ROS	NFkB/transcr. regulated under hypoxia	up	HIF1A	1.490681577	5.37586E-05	0.00065048
	ROS	NFkB	up	HMOX1	1.085687217	0.013562062	0.035674119
	UPR/ER stress	UPR target gene	down	EPHB1	-1.803718817	1.01655E-06	4.10009E-05
	UPR/ER stress	UPR target gene	up	HERPUD1	1.015708212	0.000245846	0.00165263

H1437 lung CDX

	Process (Functional gene grouping)	Upstream Pathway/Transcription Factor	expressionCond1	ID	logFC	P.Value	adj.P.Val
PAC vs d0	apoptosis	p53/NFkB	down	TRAF1	-2.005655965	9.69293E-07	3.90948E-05
	apoptosis	p53/NFkB	down	BCL2	-1.617170634	3.56854E-06	0.000107948
	apoptosis	p53/NFkB	down	TP53AIP1	-1.778741559	6.33584E-06	0.000127773
	apoptosis	p53/NFkB	up	IER3	1.348605002	1.26163E-05	0.000218081
	apoptosis	NFkB	down	ALOX12	-1.341068964	0.000504107	0.003258684
	apoptosis	NFkB	down	NOS2A	-2.163061512	0.000585419	0.00346564
	apoptosis	p53	down	BCL2L14	-1.073715416	0.0007265	0.00399575
	cell cycle		down	TP73	-2.32592196	8.18118E-09	9.89923E-07
	cell cycle	p53/NFkB	down	TERT	-1.818063518	6.28131E-06	0.000127773
	cell cycle	p53	down	CCNG1	-1.389179567	2.40452E-05	0.000363684
	DNA damage/repair	HIF1a	down	XPA	-1.193623749	4.6059E-05	0.000619238
	DNA damage/repair	prosurvival signals and oncogenes	down	BLM	-1.298156571	5.45866E-05	0.000660498
	DNA damage/repair	p53	down	SESN1	-1.057576028	9.77233E-05	0.001074956
	p38/JNK	p38	up	PHLDA1	1.012846155	0.000248105	0.002309285
	ROS	NFkB/transcr. regulated under hypoxia	up	HIF1A	1.180760224	0.000601475	0.00346564
	UPR/ER stress	UPR target gene	down	EPHB1	-1.981288875	2.68508E-07	1.62447E-05
	UPR/ER stress	UPR target gene	down	PLA2G6	-1.019594051	0.000325182	0.002481483
MF vs d0	apoptosis	p53/NFkB	down	BCL2	-1.960611405	2.46702E-07	1.49255E-05
	apoptosis	p53/NFkB	up	IER3	1.594328692	1.41718E-06	4.28698E-05
	apoptosis	NFkB	down	BCL2L1	-1.314326066	0.000144866	0.001433282
	apoptosis	p53/NFkB	down	TP53AIP1	-1.210185287	0.000478411	0.002893389
	apoptosis	NFkB	down	NOS2A	-1.542577832	0.008384952	0.02601485
	cell cycle		down	TP73	-1.860393869	2.42734E-07	1.49255E-05
	cell cycle	p53/NFkB	down	TP63	-1.35710972	0.000150902	0.001433282
	cell cycle	p53/NFkB	down	TERT	-1.374074016	0.000165834	0.001433282
	cell cycle	p53	down	CCNA2	-1.021728382	0.005285863	0.019381499
	DNA damage/repair	p53/NFkB	down	TP53I3	-1.529581821	8.14596E-06	0.000123208
	DNA damage/repair	HIF1a	down	XPA	-1.232022901	3.18621E-05	0.000428368
	DNA damage/repair	transcr. inducable by irradiation	down	XRCC3	-1.031817735	0.000162619	0.001433282
	DNA damage/repair	prosurvival signals and oncogenes	down	BLM	-1.088122797	0.000354517	0.00225771
	p38/JNK	p38	up	PHLDA1	1.485114584	2.72921E-06	6.60468E-05
	p38/JNK	p38	up	FOSL1	1.511246682	4.05091E-06	8.16934E-05
	p38/JNK	p38	up	MAFF	1.478042154	6.70678E-06	0.000115931
	p38/JNK	p38	up	OAS1	1.19449399	0.000526071	0.002893389
	ROS	NFkB/transcr. regulated under hypoxia	up	HIF1A	1.490681577	5.37586E-05	0.00065048
	ROS	NFkB	up	HMOX1	1.085687217	0.013562062	0.035674119
	UPR/ER stress	UPR target gene	down	EPHB1	-1.803718817	1.01655E-06	4.10009E-05
	UPR/ER stress	UPR target gene	up	HERPUD1	1.015708212	0.000245846	0.00165263

Primary human ovarian cancer

	Process (Functional gene grouping)	Upstream Pathway/Transcription Factor	expressionCond1	ID	logFC	P.Value	adj.P.Val
PAC vs d0	apoptosis	p53/NFkB	up	BCL2A1	2.497867992	5.3E-06	6.74825E-05
	apoptosis	p53	down	BCL2L14	-1.346953455	5.4E-06	6.74825E-05
	apoptosis	NFkB/p53	down	BCL2	-1.547595207	6.9E-06	7.41686E-05
	apoptosis	p53/NFBkB	down	TNFRSF10C	-1.88394435	4.3E-05	0.000336453
	apoptosis	NFBkB	up	ALOX12	1.231788946	0.00014	0.000938375
	apoptosis	p53/NFBkB	up	TNFRSF10D	1.656011791	0.00054	0.002697508
	apoptosis	prosurvival signals and oncogenes	down	MCL1	-1.271420996	0.00147	0.00637863
	apoptosis	p53	down	BOK	-1.54807767	0.00253	0.008858494
	apoptosis	p53	down	AIFM2	-1.389081402	0.01295	0.036144035
	apoptosis	p53	up	BID	1.60577072	0.0132	0.036144035
	DNA damage and repair	p53	down	SESN1	-1.103777569	9.8E-06	9.45751E-05
	DNA damage and repair	p53/BRCA1/AP1	down	DDB2	-1.577525339	1.1E-05	0.000101612
	DNA damage and repair	p53/NFBkB	down	TP53I3	-1.219450194	0.00056	0.002708023
	p38/JNK	p38	down	ZFP36	-3.740621121	5.7E-08	2.39049E-06
	p38/JNK	NFkB/p38	down	EGR1	-3.218010563	5.2E-07	1.09336E-05
	p38/JNK	p53/p38	down	FOS	-3.656475621	3.5E-06	5.46255E-05
	p38/JNK	p38	down	KLF6	-1.994997011	7.1E-06	7.41686E-05
	p38/JNK	p38	down	JUN	-2.323023863	3.9E-05	0.000327878
	proliferation/cell cycle	p53	down	BTG2	-1.941150382	5E-05	0.000368495
	proliferation/cell cycle	p53	down	TAp73	-1.13745292	0.00038	0.002136084
	proliferation/cell cycle	P53	up	MET	2.239782463	9.5E-07	1.70356E-05
	ROS	NFkB	up	SOD2	2.669122427	5.1E-08	2.39049E-06
	ROS	NFkB/transcriptionally regulated under hypoxia	up	HIF1A	1.340615279	2.9E-07	9.2002E-06
	UPR/ER stress	UPR target gene	down	P2RY13	-3.967102064	2.6E-08	2.39049E-06
	UPR/ER Stress	UPR target gene	down	PLA2G6	-2.117862586	4.1E-07	1.04367E-05
MF vs d0	apoptosis	p53	up	BID	1.948010859	0.00191	0.015065792
	apoptosis	p53/NFBkB	up	TNFRSF10D	1.366213542	0.00245	0.015835591
	apoptosis	p53/NFBkB	down	TNFRSF10C	-1.181590608	0.00278	0.016683948
	apoptosis	p53	down	AIFM2	-1.4107915	0.00546	0.029887122
	DNA damage and repair	p53	down	SESN1	-1.163423685	1.7E-06	7.17468E-05
	p38/JNK	p38/NFkB	down	EGR1	-3.006268061	9.7E-07	6.12749E-05
	p38/JNK	p38	down	KLF6	-2.002300567	6.7E-06	0.000151033
	p38/JNK	p38	down	ZFP36	-2.707360488	7.2E-06	0.000151033
	p38/JNK	p53/p38	down	FOS	-3.179653737	1.6E-05	0.000282371
	p38/JNK	p38	down	JUN	-1.985817176	0.00012	0.001416227
	proliferation/cell cycle	p53	up	MET	1.712337971	2E-05	0.000283739
	proliferation/cell cycle	p53	down	BTG2	-2.014378658	2E-05	0.000283739
	ROS	NFkB	up	SOD2	1.418166346	0.00036	0.00373487
	UPR/ER stress	UPR target gene	down	P2RY13	-3.402881578	3.7E-08	4.65838E-06
	UPR/ER Stress	UPR target gene	down	PLA2G6	-1.816476504	2.8E-06	8.68389E-05
